# Multiple forms of discrimination and obsessive-compulsive disorder: a prospective cohort study

**DOI:** 10.1186/s13034-025-00864-x

**Published:** 2025-02-18

**Authors:** Jason M. Nagata, Jonanne Talebloo, Thang Diep, Joan Shim, Abubakr A. A. Al-Shoaibi, Kyle T. Ganson, Alexander Testa, Jinbo He, Jason M. Lavender, Fiona C. Baker

**Affiliations:** 1 Department of Pediatrics, University of California, San Francisco, 550 16th Street, 4th Floor, Box 0503, San Francisco, CA 94143 USA; 2 Factor-Inwentash Faculty of Social Work, University of Toronto, 246 Bloor St W, Toronto, ON M5S 1V4 Canada; 3 Department of Management, Policy and Community Health, University of Texas Health Science Center at Houston, 1200 Pressler Street, Houston, TX 77030 USA; 4 Division of Applied Psychology, School of Humanities and Social Science, The Chinese University of Hong Kong, Shenzhen, 518172 Guangdong China; 5 Military Cardiovascular Outcomes Research Program (MiCOR), Department of Medicine, Uniformed Services University of the Health Sciences, 4301 Jones Bridge Rd, Bethesda, MD 20814 USA; 6 The Metis Foundation, 84 NE Interstate 410 Loop # 325, San Antonio, TX 78216 USA; 7 Center for Health Sciences, SRI International, 333 Ravenswood Ave, Menlo Park, CA 94025 USA; 8 School of Physiology, University of the Witwatersrand, 1 Jan Smuts Ave, Johannesburg, 2000 Braamfontein South Africa

**Keywords:** Adolescent, Adolescence, Obsessive-compulsive disorder, Discrimination, Racism, Prejudice, Stigma

## Abstract

**Background:**

Discrimination increases the risk for adverse mental health in minority populations, with studies showing elevated rates of obsessive-compulsive disorder (OCD) in Black adults facing racial discrimination. Yet, there is a lack of longitudinal research on the different forms of discrimination in relation to OCD risk in early adolescence. The objective of this study was to examine the prospective associations between multiple forms of discrimination and OCD in a national sample of U.S. early adolescents.

**Methods:**

We examined prospective cohort data from Year 2 (2018–2020, ages 10–13) and Year 3 (2019–2021) of the Adolescent Brain Cognitive Development Study (*N* = 7,983). Multiple logistic regression models were used to analyze associations between Year 2 past 12-month experiences of discrimination (based on race and ethnicity, country of origin, sexual orientation, weight, and combined multi-discrimination) and Year 3 probable OCD (Child Behavior Checklist; based on dichotomized t-score indicating high risk), adjusting for theoretically relevant covariates including age, sex, sexual orientation, race and ethnicity, country of origin, household income, parent education, depression, body mass index category, study site, and Year 2 probable OCD.

**Results:**

Adjusting for all covariates, multi-discrimination (OR = 1.67; 95% CI 1.23, 2.27), racial discrimination (OR = 2.77; 95% CI 1.32, 5.80), sexual orientation discrimination (OR = 2.51; 95% CI 1.11, 5.64), and weight discrimination (OR = 2.51; 95% CI 1.13, 5.59) at Year 2 were prospectively associated with developing probable OCD at Year 3. There were no significant findings for discrimination based on country of origin.

**Conclusions:**

Early adolescents who have experienced several forms of discrimination have higher odds of developing probable OCD, suggesting the utility of screening for OCD in even younger adolescents who have encountered discrimination. Educators can play a role in guiding adolescents experiencing discrimination to appropriate resources for accessing mental healthcare.

**Supplementary Information:**

The online version contains supplementary material available at 10.1186/s13034-025-00864-x.

## Introduction

Discrimination has been shown to play a critical role in the development of mental health conditions among populations with marginalized identities, including adolescents. A systematic review showed that over 50% of studies investigating the relationship between racism and health outcomes among adolescents aged 12–19 years found that perceived racism was a significant predictor of negative mental health outcomes (e.g., depression, anxiety) and behavioral problems (e.g., aggression, alcohol and drug use, smoking) [1]. However, few studies have investigated discrimination in relation to obsessive-compulsive disorder (OCD) specifically. OCD is characterized by recurrent and unwanted intrusive thoughts and repetitive behaviors that an individual feels driven to perform [2]. Among adolescents, 3% are diagnosed with OCD, and 19% have OCD symptoms (e.g., cleaning, repetition, checking, aggressive thoughts) [3, 4]. OCD has a particularly early onset in life, with nearly a quarter of males with OCD displaying symptoms before age 10 [5]. Early onset of OCD during adolescence typically correlates with a more severe clinical presentation, including higher rates of psychiatric comorbidity (e.g., ADHD symptoms, bipolar disorder) [6]. OCD may have a debilitating impact on adolescent development and have lasting effects into adulthood, such as social isolation and diminished quality of life [7]. Given that 75% of lifetime mental health disorders present by 24 years of age, adolescence is an important period to study for the development of mental health disorders and provides an opportunity to promote healthy interventions [8].

Like many other psychiatric disorders, the onset and course of illness of OCD have been linked to the interaction between traumatic life events as well as genetics. One study reported findings that the presence of one or more traumatic life events was associated with increased OCD symptom severity [9]. This relationship remained significant after controlling for various key variables indicating the influential role trauma plays in OCD onset and severity. Given that discriminatory experiences can be psychologically distressing and share features with other traumatic events, discrimination may contribute to the development of OCD through similar mechanisms.

Despite research addressing the impact of discrimination on the mental health of minority populations, there are significant gaps in the literature addressing OCD, which has mainly focused on adults and racial discrimination specifically [10–12]. For example, one study of Black adults found that those who experienced racial discrimination (versus non-racial discrimination) were more likely to develop OCD symptoms [13]. Another study of Black young adults found that an increased frequency of racial discrimination was linked to greater OCD symptom distress over time, even after accounting for factors such as age, gender, socioeconomic status, and initial levels of psychological distress [10]. However, there is a scarcity of research on discrimination and OCD in early adolescence, a crucial developmental stage marked by identity formation, concerns with peer perception, a strong desire for belonging, and sensitivity to rejection [14]. Experiencing discrimination based on social characteristics and identities may then increase the vulnerability of early adolescents to maladaptive coping mechanisms, which may include obsessions and compulsions as adolescents attempt to regain a sense of control. Additionally, there is more limited data regarding discrimination based on other social characteristics and identities, such as weight and sexual orientation discrimination. Although studies have shown significant associations between perceived weight stigma and sexual orientation discrimination with adverse mental health [15], data specific to OCD in adolescents are lacking.

The present investigation used data from Years 2 and 3 of the Adolescent Brain and Cognitive Development (ABCD) Study to address gaps in the literature by investigating the prospective association between several forms of discrimination (based on race and ethnicity, country of origin, sexual orientation, and body weight) and probable OCD, adjusting for theoretically relevant covariates. Based on prior literature, it was hypothesized that all forms of discrimination would be associated with a greater prospective risk for probable OCD in a large, nationally diverse sample of early adolescents.

## Methods

The ABCD Study consists of 11,868 children from 21 sites across the United States initially recruited in 2016–2018 (baseline) and is considered the largest longitudinal study of adolescent health and brain development. Participants were primarily recruited from school systems, which were selected based on gender, race and ethnicity, socioeconomic status, and urbanicity [16]. The ABCD Study employed probability sampling of U.S. schools within 21 catchment areas which were geographically distributed to the four major U.S. regions (West, Midwest, South, and Northeast). The ABCD Study sample was designed to ensure sufficient power to detect small to medium effects over the study’s duration, accounting for anticipated attrition [16]. Further details on the ABCD Study sample, protocol, recruitment, and measures have been described previously [16]. In Year 2, participants were predominantly 11–12 years old. Complete-case analysis wasused for missing data. There were 7,983 participants who had complete data and were included in the current analysis. Appendix A presents a comparison of sociodemographic factors, discrimination, and probable OCD in those included and excluded in the current study. Centralized institutional review board (IRB) approval was granted by the University of California, San Diego, and each respective study site. Participants provided written assent, and caregivers provided written informed consent.

### Exposure

#### Discrimination

Discrimination was assessed using a single item from the 2006 Boston Youth Survey adapted to address four separate types of discrimination experienced over the past year [16, 17]. Specifically, participants were asked, “In the past 12 months, have you felt discriminated against because of your: 1) race, ethnicity, or color; 2) country of origin; 3) sexual orientation; or 4) weight?” and responded with “Yes” or “No” to each form of discrimination. Additionally, a multi-discrimination score was created by summing all four responses (range 0 to 4).

### Outcome

#### Probable obsessive-compulsive disorder (OCD)

The Child Behavior Checklist (CBCL) is a widely used screening tool for school-aged children. This measure consists of 112 items asking a parent and/or caretaker about psychiatric symptoms and behavior problems in adolescents [18, 19]. The 2007 8-item Obsessive-Compulsive Problems was used in this investigation [20, 21], for which parents and/or caregivers responded to statements about their child’s behavior over the past six months on a scale from 0 (not true) to 2 (very true/often true). Example items include: “Repeats certain acts over and over; compulsions,” “Feels he/she might think or do something bad,” and “Can’t get his/her mind off certain thoughts; obsessions.” T-scores were calculated based on the CBCL scoring rubric [18]. Based on previous literature, a t-score of 70 or more is considered particularly high risk, and this score was used to create a binary variable for probable presence versus absence of OCD [22]. The OCD subscale has excellent internal consistency (Cronbach’s alpha = 0.84). The OCD subscale cutoff demonstrates moderate sensitivity (75.3–84.9%) and high specificity (82.2–92.5%) compared to a gold-standard clinical diagnosis of OCD, with a predictive association with DSM symptoms ranging from 70.5 to 83.3%, and a negative predictive value between 88.2 and 91.6% [21].

### Covariates

Sociodemographic covariates recorded at baseline (Year 0) were sex assigned at birth (female or male), race and ethnicity (White, Latino/Hispanic, Black, Asian/Pacific Islander, Native American, and other), and country of origin (born in the U.S. vs. born outside the U.S.) [23]. Age (years), sexual orientation (heterosexual, gay/bisexual, maybe gay/bisexual, don’t understand the question, and decline to answer), household income ($24,999 or less; $25,000 to $49,999; $50,000 to $74,999; $75,000 to $99,999; $100,000 to $199,999; and $200,000 and greater), and highest parent education (high school or less vs. college or more) were collected at Year 2. Height and weight were measured three times by a trained research assistant at Year 2, which was converted to age- and sex-specific BMI percentiles according to the Centers for Disease Control and Prevention (CDC) guidelines in adolescents [24]. Body mass index (BMI) categories (underweight, healthy weight, overweight, and obesity) were also defined based on CDC guidelines [25]. Literature suggests that childhood depression, conduct disorder, and physical abuse are associated with OCD [26, 27], indicating that these factors could be potential confounders. The CBCL Syndrome Scale Scores were used to assess for depression. Kiddie Schedule for Affective Disorders and Schizophrenia (KSADS-5), a widely used diagnostic tool for evaluating symptoms of child and adolescent mental health disorders in the DSM-5, was used to assess for conduct disorder [28, 29]. Caregivers reported experiences of the participant’s adverse childhood experiences related to physical abuse at baseline. Experiencing physical abuse included either being “shot, stabbed, or beaten brutally by a grown up in the home” or “beaten to the point of having bruises by a grown up in the home.” The study site was also included as a covariate, as was the CBCL-derived dichotomized OCD variable from Year 2.

### Statistical analysis

Data analysis was performed using Stata 18 (StataCorp, College Station, TX). The mean, standard deviation (SD), and percentages of each variable were calculated as part of descriptive statistics. Three series of multiple logistic regression models were used to estimate the associations between exposure to multiple forms of discrimination in Year 2 with probable OCD in Year 3. Model 1 was the unadjusted model without the inclusion of covariates. Model 2 adjusted for sociodemographic covariates (age, sex, race and ethnicity, household income, parent education, country of origin, and sexual orientation status), depression, study site, and OCD at Year 2. Model 3 additionally adjusted for BMI category. The Hosmer-Lemeshow goodness-of-fit test was used to determine the best-fit models [30]. We applied the Benjamini-Hochberg procedure to adjust for a false discovery rate (5%) and to account for multiple statistical tests [31]. Sample weights were applied based on the American Community Survey from the U.S. Census. Statistical significance was based on a two-sided *p* < 0.05.

## Results

The sociodemographic characteristics of participants included in the current investigation (*N* = 7983) are reported in Table 1.Participants had an average age of 12 years (SD = 0.660). Just over half of participants (51.8%) were male, 41.3% were racial and ethnic minorities, 52.3% were from families with a household income of less than $75,000, and most (96.5%) were born inside the U.S. A majority of adolescents identified as heterosexual (83.8%) and were classified as healthy weight (63.0%). At year 2, 2.6% of adolescents had depression. Less than 5% of adolescents reported feeling discriminated against for each type of discrimination based on their race and ethnicity, country of origin, sexual orientation, or weight. Among the participants, 3.1% had probable OCD in Year 2 and 3.3% had probable OCD in Year 3.

**Table 1:** Sociodemographic characteristics of Adolescent Brain Cognitive Development (ABCD) Study participants (*N* = 7,983)

Sociodemographic characteristics (baseline)	Mean (SD) / %
Age (years)	12.02 (0.66)
Sex (%)	
Female	48.2%
Male	51.8%
Race and ethnicity (%)	
White	58.7%
Latino / Hispanic	18.2%
Black	13.7%
Asian	5.1%
Native American	3.1%
Other	1.2%
Household income (%)	
$24,999 or less	14.6%
$25,000 to $49,999	19.7%
$50,000 to $74,999	18.0%
$75,000 to $99,999	14.5%
$100,000 to $199,999	25.0%
$200,000 and greater	8.2%
Parent’s highest education (%)	
High school education or less	12.8%
College education or more	87.2%
Country of origin (%)	
Born in the U.S.	96.5%
Born outside the U.S.	3.5%
Sexual orientation (%)	
Heterosexual	83.8%
Gay/bisexual	8.1%
Maybe gay/bisexual	5.5%
Don’t understand the question	1.3%
Decline to answer	1.3%
Depression (%) ^a^	2.6%
Conduct disorder (%) ^b^	1.3%
Physical abuse during childhood (%)	0.8%
BMI category (%) ^b^	
Underweight	3.4%
Healthy weight	63.0%
Overweight	16.4%
Obesity	17.1%
In the past 12 months, have you felt discriminated against because of your: (%)	
Race, ethnicity, or color	4.5%
Country of origin	1.6%
Sexual orientation	4.9%
Weight	4.9%
Multiple identities (mean (SD))	0.16 (0.50)
Probable OCD (%) ^a^	
Year 2	3.1%
Year 3	3.3%

ABCD sample weights were applied based on the American Community Survey from the U.S. Census. SD = standard deviation

^a^ Define by a t-score ≥ 70 from the Child Behavior Checklist

^b^ Based on the Kiddie Schedule for Affective Disorders and Schizophrenia

^c^ As defined by the Centers for Disease Control and Prevention

Table 2 reports the results of unadjusted and adjusted logistic regressions examining the prospective association between multiple forms of discrimination and probable OCD. In the fully adjusted models (Model 3), past 12-month experiences of racial discrimination (OR = 2.77; 95% CI = 1.32,5.80), sexual orientation discrimination (OR = 2.51; 95% CI = 1.11,5.64, and weight discrimination (OR = 2.51; 95% CI = 1.13,5.59) at Year 2 were significantly associated with greater odds of probable OCD at Year 3. Moreover, experiencing a greater number of discrimination types (i.e., multi-discrimination) at Year 2 was significantly associated with greater odds of probable OCD at Year 3 (OR = 1.67; 95% CI = 1.23,2.27) in the fully adjusted model. Finally, in the fully adjusted model, discrimination based on country of origin at Year 2 was not significantly associated with probable OCD at Year 3 (OR = 2.32; 95% CI = 0.53,10.18). Appendix B shows Hosmer-Lemeshow goodness-of-fit test statistics for models presented in Table 2. Overall, models 1 and 2 show they are good fits since the Pearson chi-square statistics are fairly low.

**Table 2:** Multi-discrimination associations with OCD in the Adolescent Brain Cognitive Development (ABCD) Study

	Probable OCD					
	Model 1 (*N* = 7,983)		Model 2 (*N* = 7,983)		Model 3 (*N* = 5,793)	
	OR (95% CI)	*p*	OR (95% CI)	*p*	OR (95% CI)	*p*
In the past 12 months, have you felt discriminated against because of your:						
Race, ethnicity, or color	**2.36 (1.44**,** 3.87)**	**0.001**	**2.46 (1.28**,** 4.72)**	**0.007**	**2.77 (1.32**,** 5.80)**	**0.007**
Country of origin	**2.28 (1.04**,** 5.01)**	**0.040**	2.64 (0.89, 7.78)	0.079	2.32 (0.53, 10.18)	0.264
Sexual orientation	**3.18 (2.04**,** 4.97)**	**< 0.001**	**2.18 (1.06**,** 4.46)**	**0.033**	**2.51 (1.11**,** 5.64)**	**0.027**
Weight	**3.51 (2.26**,** 5.47)**	**< 0.001**	**2.36 (1.25**,** 4.48)**	**0.008**	**2.51 (1.13**,** 5.59)**	**0.024**
Sum score	**1.80 (1.51**,** 2.13)**	**< 0.001**	**1.64 (1.26**,** 2.14)**	**< 0.001**	**1.67 (1.23**,** 2.27)**	**0.001**

Bold indicates statistical significance after the Benjamini-Hochberg procedure. OR = odds ratio. Models represent the abbreviated output from logistic regression. Model 1 is an unadjusted model. Model 2 adjusts for sociodemographic characteristics, including age, sex, race and ethnicity, household income, parent education, country of origin, sexual orientation status, depression, conduct disorder, physical abuse during childhood, study site, and OCD at year 2. Model 3 additionally adjusts for BMI category. Sample weights from the Adolescent Brain Cognitive Development Study were applied based on the American Community Survey from the U.S. Census.

## Discussion

Largely consistent with the hypothesis, the results of this investigation in a demographically diverse national sample of U.S. early adolescents found that discrimination based on race and ethnicity, sexual orientation, and weight, as well as multi-discrimination, were prospectively associated with significantly greater odds of probable OCD at Year 3, adjusting for Year 2 probable OCD and numerous theoretically relevant covariates. In contrast to the hypothesis, there was no significant prospective association between country-of-origin discrimination and probable OCD, although the smaller subsample of adolescents born outside the U.S. may have limited the statistical power of that model.

The significant prospective relationship between discrimination and probable OCD in our study of young adolescents is consistent with previous research showing that the different forms of discrimination may be linked with poorer mental health outcomes in adults [10, 23]. One study of Black adults found that OCD symptoms of obsession (e.g., contamination, unacceptable thoughts) and compulsion (e.g., washing and checking, arranging) were more prevalent amongst those who experienced racial discrimination (versus non-racial discrimination) [13]. Research also suggests that more frequent racial discrimination was prospectively associated with distress associated with obsessive-compulsive symptoms among young adults [12]. While no prior work examined the associations between other forms of discrimination and OCD either in adolescents or adults to our knowledge, there is evidence of association with other psychiatric disorders [15]. For example, one study found that those experiencing perceived weight discrimination (versus not) were 2.41 times more likely to have more than three psychiatric diagnoses [32]. Our findings contribute to the literature by highlighting that discrimination is a predictor of probable OCD even among early adolescents, an under-researched age group that is susceptible to the onset and exacerbation of various forms of adverse mental health [33].

Minority Stress Theory may inform potential mechanisms by which racial, sexual orientation, and weight discrimination increase the risk of probable OCD in early adolescence. Specifically, discrimination and stigmatization based on marginalized identities and characteristics may increase stress that in turn contributes to adverse health behaviors and psychological outcomes [34, 35]. For example, one study of lesbian young adults found that discrimination and sexual minority stress were associated with higher social anxiety [36], which in turn was associated with increased body shame and binge eating [37]. Another population-based study of Black adults proposed that navigating chronic racial discrimination may contribute to OCD development by exhausting the psychological resources needed to manage obsessions and/or compulsions [13].

Notably, we also found that experiencing more forms of discrimination was associated with a greater prospective risk for probable OCD. This finding is consistent with prior research on the mental health of communities that face more than one type of discrimination. For example, one study found that multifactorial discrimination, or the total discrimination attributed to more than one social identity (e.g., sexual orientation, race and ethnicity, gender), predicted elevated depression scores, lower psychological well-being, and greater odds of substance use disorder diagnosis [38]. Another study using a multivariate model that included both racial and sexual orientation discrimination found an additive effect in predicting depressive symptoms among Black sexual minority adolescents [32]. Taken together, our study thus further suggests the importance of considering an intersectional framework that takes into account interactions between multiple marginalized social identities in examining the impact of discrimination on mental health outcomes [40].

The one form of discrimination that was not significantly, prospectively associated with probable OCD after adjusting for covariates was country-of-origin discrimination. One potential explanation is that adolescents perceive this type of discrimination differently or may find it difficult to separate discrimination based on their country of origin from other aspects of their identity, such as their race and ethnicity [40], which may be more salient. Notably, given that it was also the least common type of discrimination, it is also possible that the analysis with this variable had lower power to detect statistically significant effects. Country-of-origin discrimination may also be less relevant within the timeframe examined in our study but could be more relevant five years later, during high school or college.

### Strengths and limitations

The current investigation had several strengths. To our knowledge, this is the first study to examine the association between discrimination and probable OCD using prospective data from a large, national, and demographically diverse sample of early adolescents. Additionally, we examined several forms of discrimination separately as well as in combination (i.e., multi-discrimination). However, there were also limitations, including the reliance on self-reported data for discrimination and parent-reported data for obsessive-compulsive symptoms, which may introduce bias into the results. The discrimination measure for each type was based on one dichotomous question and did not allow for an examination of the frequency or severity of discrimination in relation to risk for probable OCD. Although we adjusted for several potential confounders including sociodemographic, mental health, and childhood trauma variables based on prior literature, there is the possibility of unmeasured confounders. It is possible that the lower prevalence of country of origin discrimination could lead to smaller cell sizes and higher statistical error, so those results should be interpreted cautiously. Finally, future studies should investigate other predictors of OCD development in adolescents to provide a more comprehensive understanding of the risk factors of OCD and their interactions to inform predictive models.

## Conclusions

In this study, early adolescents who experienced discrimination based on race and ethnicity, sexual orientation, and/or weight in the previous 12 months showed higher prospective odds of having probable OCD one year later. These findings suggest that screening for OCD symptoms should be considered for even younger adolescent patients who have marginalized identities and may have encountered discrimination. Educators can also play an essential role by referring adolescents experiencing discrimination for mental health screening and treatment. Moreover, to address stigmatization and discrimination based on social identities and characteristics (e.g. race and ethnicity, sexual orientation, weight) that adolescents may face at school [41], schools can consider bolstering protective factors such as inclusive curricular and anti-bullying guidelines that specifically focus on discrimination, which can cultivate school connectedness and perceptions of personal safety [42–44]. These strategies could reduce stress stemming from the discrimination faced by early adolescents with marginalized identities and, in turn, may lower the risk of adverse mental health outcomes, including OCD. Future research should further examine the specific mechanisms by which discrimination may impact the development or exacerbation of OCD in younger adolescents, and take into account the multiple marginalized and intersectional identities of adolescents when investigating discrimination and mental health outcomes.

## Electronic supplementary material

Below is the link to the electronic supplementary material.

Supplementary Material 1

## Data Availability

Data used in the preparation of this article were obtained from the ABCD Study (https://abcdstudy.org), held in the NIMH Data Archive (NDA).

## Abbreviations

ABCD: Adolescent Brain Cognitive Development Study
CBCL: Child behavior checklist
OCD: Obsessive-compulsive disorder
DSM: Diagnostic and statistical manual of mental disorders
