# Supplementary material for: Multiple forms of discrimination and obsessive-compulsive disorder: a prospective cohort study
Source: Child Adolesc Psychiatry Ment Health. 2025 Feb 18;19:11. doi: 10.1186/s13034-025-00864-x (PMC11837406; doi:10.1186/s13034-025-00864-x)
Supplement: Supplementary file 1 — Supplementary Material 1 [file 13034_2025_864_MOESM1_ESM.docx]

| Appendix A. Inclusion and Exclusion of Adolescent Brain Cognitive Development (ABCD) Study participants (N=11,868) | | | |
| --- | --- | --- | --- |
|  | **Included (N=7,983)** | **Excluded (N=3,885)** | **p** |
| **Sociodemographic characteristics** | **Mean (SD) / %** | **Mean (SD) / %** |  |
| Age (years) | 12.02 (0.660) | 12.00 (0.672) | 0.488 |
| Sex (%) |  |  | 0.420 |
| Female | 48.2% | 49.3% |  |
| Male | 51.8% | 50.7% |  |
| Race and ethnicity (%) |  |  | <0.001 |
| White | 58.7% | 39.8% |  |
| Latino / Hispanic | 18.2% | 25.3% |  |
| Black | 13.7% | 22.2% |  |
| Asian | 5.1% | 7.0% |  |
| Native American | 3.1% | 3.5% |  |
| Other | 1.2% | 2.1% |  |
| Household income (%) |  |  | <0.001 |
| $24,999 or less | 14.6% | 24.4% |  |
| $25,000 to $49,999 | 19.7% | 21.4% |  |
| $50,000 to $74,999 | 18.0% | 17.9% |  |
| $75,000 to $99,999 | 14.5% | 12.1% |  |
| $100,000 to $199,999 | 25.0% | 19.6% |  |
| $200,000 and greater | 8.2% | 4.6% |  |
| Parent's highest education |  |  | <0.001 |
| High school education or less | 12.8% | 23.9% |  |
| College education or more | 87.2% | 76.1% |  |
| Country of origin |  |  | 0.045 |
| Born in the U.S. | 96.5% | 95.2% |  |
| Born outside the U.S. | 3.5% | 4.8% |  |
| Sexual Orientation (%) |  |  | <0.001 |
| Heterosexual | 83.8% | 77.5% |  |
| Gay/bisexual | 8.1% | 11.4% |  |
| Maybe gay/bisexual | 5.5% | 5.5% |  |
| Don't understand the question | 1.3% | 3.2% |  |
| Decline to answer | 1.3% | 2.4% |  |
| Depression (%) ^a^ | 2.6% | 3.5% | 0.052 |
| Conduct disorder (%) ^b^ | 1.3% | 1.0% | 0.270 |
| Physical abuse during childhood (%) | 0.8% | 1.0% | 0.328 |
| BMI category (%) ^b^ |  |  | <0.001 |
| Underweight | 3.4% | 3.4% |  |
| Healthy weight | 63.0% | 55.5% |  |
| Overweight | 16.4% | 19.1% |  |
| Obesity | 17.1% | 22.0% |  |
| In the past 12 months, have you felt discriminated against because of your: (%) |  |  |  |
| Race, ethnicity, or color | 4.5% | 6.3% | 0.008 |
| Country of origin | 1.6% | 3.5% | <0.001 |
| Sexual orientation | 4.9% | 8.2% | <0.001 |
| Weight | 4.9% | 8.2% | <0.001 |
| Multiple identities (mean (SD)) | 0.16 (0.502) | 0.20 (0.574) | 0.060 |
| Probable OCD (%) ^a^ |  |  |  |
| Year 2 | 3.1% | 4.3% | 0.030 |
| Year 3 | 3.3% | 3.8% | 0.400 |
| ABCD sample weights were applied based on the American Community Survey from the US Census. SD = standard deviation | | | |
| ^a^ Define by a t-score ≥70 from the Child Behavior Checklist  ^b^ Based on the Kiddie Schedule for Affective Disorders and Schizophrenia |  |  |  |
| ^c^ As defined by the Centers for Disease Control and Prevention. | |  |  |

| Appendix B. Goodness-of-fit tests for multi-discrimination associations with OCD in the Adolescent Brain Cognitive Development (ABCD) Study | | | | | | |
| --- | --- | --- | --- | --- | --- | --- |
|  | Probable OCD | | | | | |
|  | Model 1 (N=7,983) | | Model 2 (N=7,983) | | Model 3 (N=5,793) | |
|  | F statistic | p | F statistic | p | F statistic | p |
| In the past 12 months, have you felt discriminated against because of your: |  |  |  |  |  |  |
| Race, ethnicity, or color | 0.00 | 1.000 | 0.83 | 0.593 | **1.90** | **0.047** |
| Country of origin | 0.00 | 1.000 | 0.76 | 0.656 | **594.32** | **<0.001** |
| Sexual orientation | 0.00 | 1.000 | 0.79 | 0.627 | 1.39 | 0.184 |
| Weight | 0.00 | 1.000 | 1.00 | 0.437 | **2.34** | **0.013** |
| Sum score | 0.00 | 1.000 | 1.40 | 0.183 | **597.09** | **<0.001** |
| Bold indicates p<0.05. Models represent the abbreviated output from the Hosmer–Lemeshow goodness-of-fit test. A good fit model has a non-significant p-value. Model 1 is an unadjusted model. Model 2 adjusts for sociodemographic characteristics, including age, sex, race and ethnicity, household income, parent education, country of origin, sexual orientation status, depression, study site, and OCD at year 2. Model 3 additionally adjusts for BMI category. | | | | | | |
